# Neurotrophins and Proneurotrophins as Biomarkers for Overactive Bladder Syndrome in Aging Females

**DOI:** 10.3390/metabo15070429

**Published:** 2025-06-23

**Authors:** Claudia Covarrubias, Philippe G. Cammisotto, Lysanne Campeau

**Affiliations:** 1 Lady Davis Institute, McGill University, Montreal, QC H3T1E2, Canada; claudia.covarrubias@mail.mcgill.ca (C.C.); philippe.cammisotto.1@ulaval.ca (P.G.C.); 2 Urology Department, Jewish General Hospital, Montreal, QC H3T1E2, Canada

**Keywords:** overactive bladder, biomarkers, neurotrophins, NGF, BDNF

## Abstract

**Background/Objectives**: Overactive bladder (OAB), common in elderly women, involves urgency, frequency, and nocturia, with complex phenotypes. The use of neurotrophins as non-invasive urinary biomarkers has been previously explored. The objective of this study was to assess the diagnostic and therapeutic utility of urinary biomarkers in a Canadian population of aging female OAB patients. **Methods**: We conducted a single-center prospective study of aging female patients diagnosed with OAB and age-matched healthy controls, where we conducted pre- and post-treatment assessments using a combination of clinical questionnaires, voiding diaries, and urinary biomarkers nerve growth factor (NGF), proform of NGF (proNGF), brain-derived neurotrophic factor (BDNF), proform of BDNF (proBDNF), and neurotrophin receptor p75 extracellular domain (p75^ECD^)) quantified using ELISA. Baseline and post-treatment urinary biomarker levels in OAB patients were compared with those of controls. **Results**: OAB patients and controls at baseline displayed significant differences in neurotrophin levels and in their ratios of mature/precursors. In the post-treatment OAB cohort, only NGF and proNGF exhibited significant improvement correlating with clinical symptom relief. Biomarkers in non-responders remained unchanged, suggesting heterogeneity in therapeutic response. **Conclusions**: Urinary neurotrophins show promise as non-invasive diagnostic markers of OAB and monitoring treatment response in aging female patients. While this study focused on patients broadly diagnosed with OAB, future research should aim to classify OAB subtypes—such as those based on urodynamic studies or underlying pathophysiology—to better understand how urinary neurotrophins can differentiate between mechanisms like detrusor overactivity, detrusor underactivity, or bladder outlet obstruction. This will enhance their relevance in guiding personalized treatment strategies and predicting outcomes.

## 1. Introduction

Overactive bladder (OAB) is a common urological condition that disproportionately affects the elderly, particularly aging women [1]. Characterized by urgency, frequency, and nocturia, OAB significantly impacts quality of life [2]. Current classifications are based on the presence of urinary incontinence (OAB-wet vs. OAB-dry) and underlying etiologies, such as neurogenic or idiopathic OAB [3,4]. While its prevalence increases with age, the complexity of OAB phenotypes also increases, as overlapping risk factors, polypharmacy, and comorbidities further blur the boundaries between differential diagnoses and clinical management [1,2,4,5]. Diagnosing and assessing treatment response is challenging, and traditional tools, like urodynamic testing, may be less reliable or poorly tolerated, emphasizing the need for alternative, non-invasive biomarkers [1]. A biomarker is defined as any measurable objective indication, ranging from simple to complex, that serves as an indicator of normal or pathological biological states in diagnostic processes or as a prognostic tool for assessing treatment response [6].

Recent research has identified neurotrophins, particularly nerve growth factor (NGF) and brain-derived neurotrophic factor (BDNF), and their precursors as urinary biomarkers for OAB. They carry a high potential to aid diagnosis, provide insight into neurogenic mechanisms, and hold promise for monitoring therapeutic responses [7,8,9,10,11]. Neurotrophins are critical modulators of neural plasticity in the bladder’s sensory and motor pathways, contributing to sensory pathway remodeling, driving symptoms like urgency and increased voiding frequency by enhancing neural excitability in OAB [12]. Elevated neurotrophin levels, particularly NGF, are implicated in detrusor overactivity and may serve as urinary biomarkers for improving OAB diagnosis and monitoring treatment efficacy [10]. Targeting neurotrophins offers a promising therapeutic strategy to mitigate OAB-related lower urinary tract symptoms (LUTS) by addressing underlying neuronal remodeling.

Building on previous findings related to NGF metabolism imbalances [13], urinary metabolomics linked to OAB severity [14], and reduced proform of BDNF (proBDNF)/BDNF ratios in OAB patients [15], our study aims to evaluate the association of urinary neurotrophin levels in OAB, specifically evaluating their potential as diagnostic and prognostic biomarkers for OAB.

## 2. Materials and Methods

### 2.1. Patient Profiles

Patient recruitment, as part of our previously published work, was described previously [11,12,13] with IRB approval by the Medical-Biomedical Research Ethics Committee (REC) of the Integrated Health and Social Services University Network for West-Central Montreal (IRB: 2016-328, 15-022, approved on 20 June 2017). A second study (IRB: 2022-3131, approved on 25 January 2022) was obtained for the recruitment of additional patients that provided baseline and post-treatment samples. All participants were interviewed in person and gave written informed consent in agreement with the Helsinki Declaration before commencing study activities.

All patients (N = 56) were recruited at the Urology department of the Jewish General Hospital, Montreal, Canada. Participants in the OAB group were women aged between 50 and 86 years old (*n* = 33) who were diagnosed with OAB (with or without prior treatment), and their symptoms included: urinary frequency and urgency, or urge incontinence, for at least 3 months. In accordance with current clinical guidelines, all participants were initially counselled on behavioral approaches, including bladder training, lifestyle modifications, and pelvic floor muscle exercises, as a first-line intervention. Only those who did not achieve adequate symptom control with conservative measures were progressed to pharmacological treatment. To reduce confounding effects, participants were required to have discontinued any pharmacological treatment for at least 3 weeks prior to the baseline measurement. A routine negative screening urine culture was also performed to exclude infection. Out of the total combined 33 OAB patients, only 10 undertook analysis at baseline and 3 months after pharmacological treatment commenced. Treatment included ß3-adrenoceptor agonist and antimuscarinic, mirabegron (25 mg and 50 mg once daily) or Solifenacin (5 mg once daily), respectively. The group of control subjects (*n* = 23) was age-matched healthy volunteers who had no urinary symptoms, no current or prior use of OAB medications, and a negative urine test for any infection. Exclusion criteria for both groups were as follows: established diabetes mellitus, history of malignancies or pelvic radiotherapy, pelvic organ prolapse, urinary tract infection, neurogenic lower urinary tract dysfunction, and hepatic or renal impairment (creatinine clearance < 70 mL/min).

### 2.2. Demographic and Clinical Differences

Complete medical history, physical examination, screening urinalysis, 1-day voiding diary, and validated symptom questionnaires were carried out on every participant. Voiding diaries allowed the estimation of 24-h, daytime, and nighttime frequencies, total 24-h voiding volume, nocturnal voiding volume, mean voided volume per micturition, and maximum voided volume. All participants completed the Overactive Bladder Symptom Score (OABSS), the International Consultation on Incontinence Questionnaire-Short Form (ICIQ-SF), and the Incontinence Impact Questionnaire (IIQ-7). Fasting glucose and insulin levels were measured to calculate the Homeostatic Model Assessment of Insulin Resistance (HOMA-IR) as an index for insulin resistance. Significant insulin resistance was determined by values above 2.9. Only 10 OAB patients completed the 3-month follow-up to evaluate post-treatment changes. These patients were further categorized as responders or non-responders, with responders defined as achieving a minimum 30% reduction in symptoms, as measured by improvements in questionnaire scores. This threshold was used to assess meaningful clinical improvement, allowing for a clear distinction between those who experienced significant symptom relief and those who did not.

### 2.3. Collection and Analysis of Urine Samples

Midstream early morning urine samples were gathered by patients in two sterile plastic containers. One was kept at 4 °C for bacterial culture. The other container was kept at −20 °C. Dietary restrictions were not requested during urine collection. Upon reception at the hospital, samples were immediately thawed, centrifuged at 10,000 rpm for 3 min to remove cells and debris, then aliquoted and stored at −80 °C until use. Pellets were kept as well. No preservatives were added as they might interfere with further analysis. Laboratory staff were unaware of which samples were OAB or controls. ELISA kits for NGF, proform of NGF (proNGF), BDNF, proBDNF, and neurotrophin receptor p75 extracellular domain (p75^ECD^) were purchased from Biosensis (Thebarton, Australia). Samples were analyzed in duplicates. Creatinine was measured using the Jaffe reaction [16].

### 2.4. Statistics

Group comparisons were conducted using both parametric and non-parametric methods. Student’s *t*-test was applied for normally distributed data, while the Mann–Whitney U test was used for non-normally distributed data. The Wilcoxon signed-rank test was conducted for paired comparisons. One-way ANCOVA was conducted to control for confounders such as age, HOMA-IR, and renal function when comparing differences between the control and OAB cohorts. Spearman’s rank correlation coefficient was performed to assess the strength of association between urinary biomarkers, questionnaire scores, voiding diary parameters, and clinical markers. Two-way ANOVA was used to evaluate the main effects of treatment and interactions between responders and non-responders, with post-hoc pairwise comparisons conducted using uncorrected Fisher’s Least Significant Difference (LSD) test. Receiver operating characteristic (ROC) curves were calculated using the Wilson/Brown method with 95% Confidence Intervals (CI). All statistical analyses were performed using GraphPad Prism Version 10.2.3 (GraphPad Software, San Diego, CA, USA). Statistical significance was defined as *p* < 0.05.

## 3. Results

### 3.1. Subject Characteristics

A total of 56 female participants successfully completed the study protocol and are included in the analysis. Concordant with our previous publications, the mean age for the OAB group was higher than the control group (69.49 ± 10.14 vs. 56.62 ± 5.46 years in controls, *p* < 0.0001). Both groups had no significant difference in the body mass index (BMI), demographics, or blood pressure. The OAB group was found to have higher HOMA-IR level (4.01 ± 3.71 vs. 2.03 ± 0.97 in controls, *p* = 0.028) and reduced renal function as expressed by estimated glomerular filtration rate (eGFR) (75.33 ± 18.08 vs. 96.39 ± 16.45 in controls, *p* = <0.0001) when compared to the control group. Additionally, OAB symptom severity, as reflected in the voiding diary and questionnaire scores, was significantly higher in the OAB group (Table 1).

### 3.2. Biochemical Urinalysis

**Figure 1:**
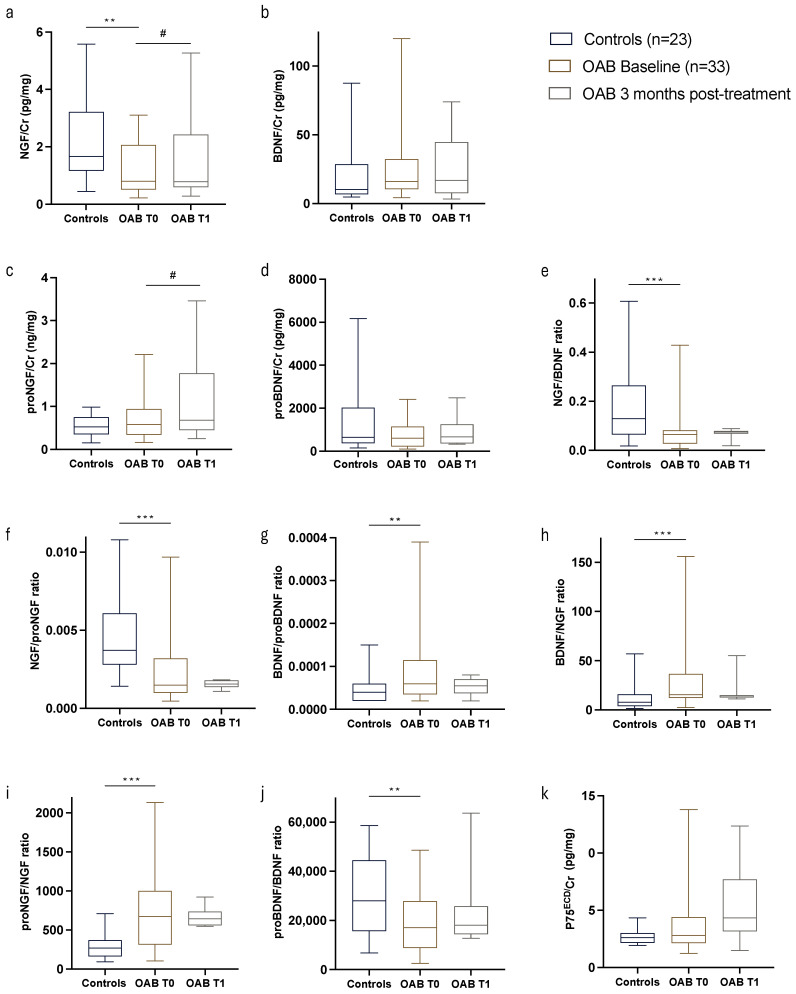
Comparison of urinary biomarker levels among controls and overactive bladder patients at baseline (T0) and 3 months post-treatment (T1). (**a**) Nerve growth factor (NGF), (**b**) Brain Derived Neurotrophic Factor (BDNF), (**c**,**d**) neurotrophin precursors (proNGF and proBDNF), (**e**–**j**) neurotrophin ratios, and (**k**) Neurotrophin receptor p75 extracellular domain (p75^ECD^) were measured and compared amongst the patient groups. ** *p* < 0.005, *** *p* < 0.0005, for Mann–Whitney U test (Controls vs. OAB T0); # *p* <0.05 for Wilcoxon paired *t* test (OAB T0 vs. OAB T1).

All variables tested were reported to urinary creatinine level, as summarized in Table 2. Biomarker levels in the urine differed significantly between control subjects and OAB patients, apart from proBDNF, BDNF, p75^ECD^, and proNGF (Figure 1). We further adjusted the urinary levels of neurotrophins and their precursor molecules with metabolic confounders, including the HOMA index, age, and estimated kidney function level (Appendix A; Table A1).

**Figure 2:**
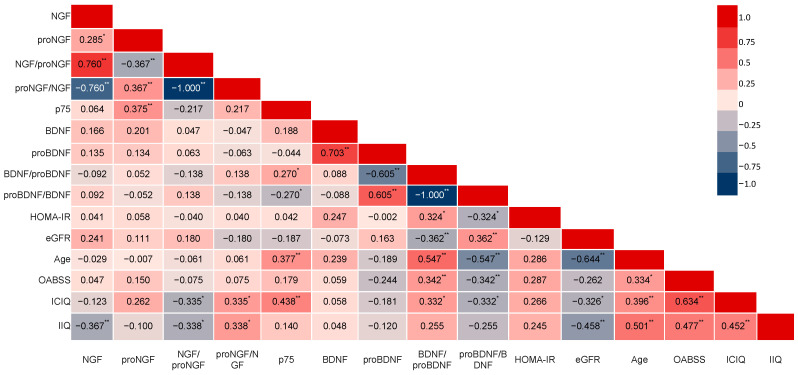
Correlation between selected demographics and questionnaires versus neurotrophins and their ratios in the total cohort. Color scale bar represents the strength of correlation; Correlation matrix based on Spearman’s rank correlation coefficient (−1 ≤ *r* ≤ 1), * *p* < 0.05 and ** *p* < 0.01 (2-tailed) for the significance of correlation.

**Figure 3:**
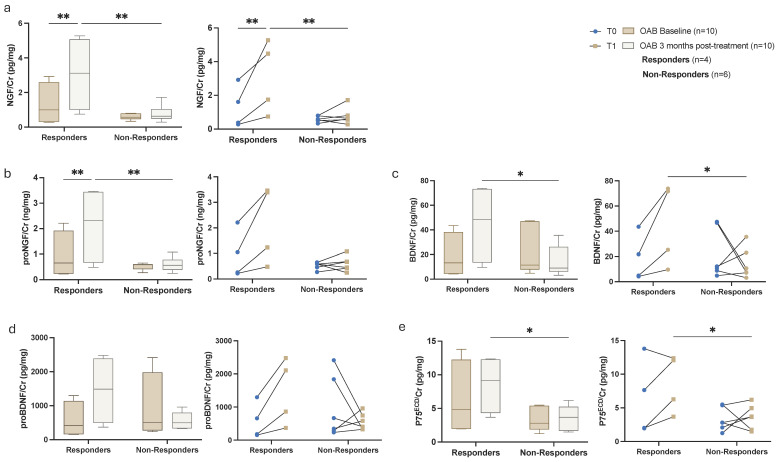
Urinary biomarker levels in OAB patients treated for 3 months, comparing responders to non-responders by group and by individual response. Biomarkers measured included: (**a**) NGF, (**b**) proNGF, (**c**) BDNF, (**d**) proBDNF, and (**e**) p75^ECD^, pre- and post-treatment. Statistical analysis was conducted using two-way ANOVA to evaluate the main effects of treatment and the interaction between groups. Post-hoc pairwise comparisons were carried out using the uncorrected Fisher’s Least Significant Difference (LSD) test. * *p* < 0.05, ** *p* < 0.005.

**Figure 4:**
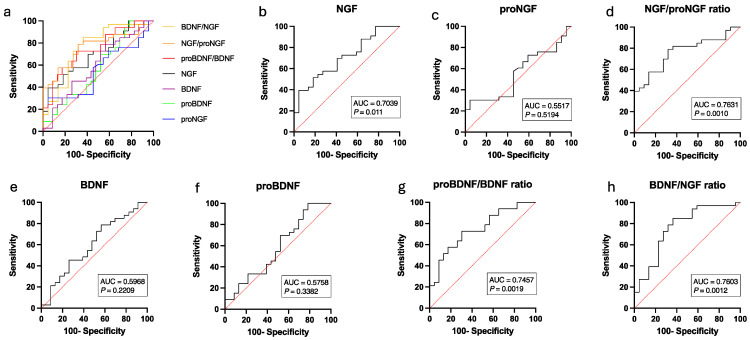
The receiver operating characteristic (ROC) analysis using the Wilson/Brown method was performed comparing control versus OAB cohorts at baseline measurements. (**a**) Ensemble area under the curve (AUC) analysis of urinary biomarkers. (**b**–**h**) Individual AUC analysis of each biomarker tested, including (**b**) NGF, (**c**) proNGF, (**d**) NGF/proNGF, (**e**) BDNF, (**f**) proBDNF, (**g**) proBDNF/BDNF, and (**h**) BDNF/NGF ratio.

The activity of NGF and its ratios proNGF/NGF and NGF/proNGF were significantly correlated to the scores of OAB symptom questionnaires (ICIQ-SF, IIQ-7) (Figure 2). Among the OAB treatment group, 4 patients (40%) exhibited symptomatic improvement (responders), whereas 6 patients (60%) did not experience such improvement (non-responders), as determined by responses to the OABSS, ICIQ, and IIQ questionnaires. When analyzing the neurotrophin levels amongst these subgroups, we found that there was a marked difference between pre- and post-treatment in the neurotrophin measurements on the responder group (Figure 3) that was not previously shown when assessing globally the OAB group (*n* = 33). ROC curve analysis revealed that the urinary NGF/proNGF ratio yielded the highest area under the curve (AUC) (AUC, 0.76, [95%CI, 0.62–0.89]; *p* = 0.0012) with a threshold cut-off of >14.50 that represented a sensitivity of 63.64% and specificity of 77.27% (Likelihood Ratio = 2.8; Youden’s J Statistic: J = 40.91) (Figure 4).

## 4. Discussion

Our study revealed significant baseline differences in urinary neurotrophin levels and their mature/precursor ratios between OAB patients and controls, with post-treatment improvements in neurotrophin levels correlating with clinical symptom relief in responders. These findings highlight the potential of urinary neurotrophins as non-invasive biomarkers for diagnosing OAB and monitoring treatment response, while the unchanged biomarker levels in non-responders underscore the heterogeneity in therapeutic outcomes, warranting further exploration of OAB subtypes to refine personalized treatment strategies. Our findings, while consistent with prior research, provide additional data that further validate the proposed biomarkers [13,14,15]. This study reinforces the relevance of neurotrophins in OAB diagnosis and management while highlighting their broader applicability in assessing treatment response. In this study, we applied the Wilcoxon matched-pairs test to assess changes in exploratory urinary biomarkers in patients with OAB from baseline to 3 months post-treatment. While several biomarkers demonstrated significant differences, suggesting a measurable response to treatment, a subset of biomarkers did not show significant changes despite the paired analysis. This may reflect inherent variability in individual responses to treatment, differences in the biological processes driving OAB, or limitations in the sensitivity and temporal responsiveness of the selected biomarkers. It is also possible that the effect of treatment on these specific biomarkers is either delayed or not pronounced within the 3-month observation period. In particular, the ratio of mature neurotrophins to proneurotrophins provided a more robust indicator of diagnostic and therapeutic outcomes compared to the standalone values of either biomarker. This ratio more accurately reflects the dynamic balance between pro-survival and pro-apoptotic pathways, highlighting its significance in assessing disease progression and treatment response in older female patients with OAB. Future studies should incorporate urodynamic studies or other investigative tools to stratify OAB phenotypes and assess the specific role of biomarkers in differentiating underlying mechanisms.

The significant differences in age and insulin resistance between the OAB and control groups likely influenced our results, as both factors are linked to OAB severity and metabolic syndrome (MetS). Peyronnet et al. have suggested that metabolic syndrome represents a distinct OAB phenotype, characterized by unique pathophysiological mechanisms [5]. Older age may exacerbate OAB symptoms due to age-related bladder changes, while insulin resistance, a hallmark of MetS, promotes inflammation and urothelial dysfunction, potentially worsening OAB and reducing treatment efficacy [17,18]. These findings suggest that MetS should be considered when interpreting OAB outcomes, and future studies should adjust for these variables to minimize confounding effects.

While this study concentrated on the role of neurotrophins in OAB, the findings may have broader implications for a range of urological and non-urological disorders where neuronal signaling and inflammation are pivotal. The existing literature highlights the multifaceted role of neurotrophins in regulating physiological, diagnostic, and therapeutic processes across various biological systems, reflecting their widespread production by numerous cell types in the human body, either constitutively or in response to specific stimuli [19,20]. Neurotrophin functions are critical during pregnancy for placental and embryonic development, as well as in postnatal life. They mediate brain-lung axis interactions, underscoring their significance in coordinating neurological and respiratory processes such as sudden infant death syndrome, asthma, congenital central hypoventilation syndrome, and bronchopulmonary dysplasia [21]. BDNF has also been shown to influence synaptic plasticity, neurogenesis, and cognitive performance [22]. In a recent meta-analysis, BDNF levels were found to be decreased in conditions such as bipolar disorder, major depressive disorder, obsessive-compulsive disorder, Parkinson’s disease, and schizophrenia compared to controls, while BDNF was increased in post-traumatic stress disorder [23]. Other neuropathologies linked to decreased BDNF levels and signaling are chronic and neuropathic pain, brain cancer, and neurodegenerative disorders, such as Alzheimer’s disease, Parkinson’s disease, and Huntington’s disease [24,25].

Our study has several limitations. The enrollment numbers were small, and we did not investigate serial changes of urinary neurotrophin levels before or after 3 months. However, Suh et al. showed that early serial measurements (e.g., at 4 weeks) do not appear to provide additional predictive value, supporting a strategy of assessing NGF/Cr at the completion of a 12-week treatment cycle to guide clinical decisions, such as whether to continue or stop antimuscarinic therapy [26].

Another key limitation of our study is the use of a one-day voiding diary rather than the three-day diary recommended by current clinical guidelines for evaluating LUTS. While this may raise concerns about the completeness and representativeness of the data, prior research has reported that patient compliance with three-day diaries can be challenging due to burden, particularly among women and those with urgency incontinence [27,28]. Notably, in the LURN (Symptoms of Lower Urinary Tract Dysfunction Research Network) study, despite aiming for three days of diary data, the authors found that fewer than 50% of diaries were complete, and approximately 39% were incomplete but usable. Importantly, they observed limited variability across the three diary days, concluding that one day of data collection might be sufficient for certain analyses, although this was not formally compared in a controlled manner [27]. Therefore, while our use of a one-day diary should be viewed as a limitation, the evidence suggests that it may still yield clinically relevant information without the added participant burden, especially noted in the female population. Another important limitation of this study is the absence of urodynamic studies (UDS) to confirm detrusor overactivity (DO) in our OAB cohort. DO is observed in approximately 33% of female OAB patients and is a key physiological feature associated with storage LUTS [29]. The inclusion of urodynamic parameters could have enhanced the homogeneity of our patient population and strengthened the correlation analysis between neurotrophin levels and specific bladder dysfunction mechanisms. However, we employed a symptom-based diagnosis of OAB, focusing on urgency, frequency, and nocturia, which reflects routine clinical practice where UDS is not universally performed due to its invasive nature, cost, and limited accessibility [30,31]. Prior studies have demonstrated elevated NGF and BDNF levels in OAB patients regardless of DO status, supporting the relevance of our findings to symptom-driven diagnosis [8]. Nevertheless, the lack of UDS may introduce variability in our cohort and limit insights into the relationship between neurotrophins and DO-specific mechanisms. Lastly, our treatment response analysis was limited due to the use of two pharmacological agents, oxybutynin (antimuscarinic) and mirabegron (β3-adrenoreceptor agonist), in the OAB treatment group. Due to the small sample size, we excluded sub-analysis by medication to avoid underpowered comparisons, focusing instead on distinguishing responders from non-responders. The distinct mechanisms of these medications may differentially affect biomarkers, particularly in OAB phenotypes like MetS, where antimuscarinics, sacral neuromodulation, and botulinum toxin are less effective due to inflammation and urothelial dysfunction [5]. Conversely, mirabegron, originally developed as an anti-obesity drug [32], shows comparable efficacy in obese and nonobese OAB patients, suggesting suitability for MetS patients, though dose adjustments may be needed. Future studies with larger cohorts are needed to clarify medication-specific effects on biomarkers.

## 5. Conclusions

In conclusion, our study highlights the complexity of OAB pathophysiology and the need for further investigation into the specific pathways these biomarkers represent. Future studies should focus on longer follow-up periods, larger and more diverse cohorts, and advanced tools for OAB subtype classification to evaluate the full spectrum of biological responses and refine the role of urinary biomarkers in guiding personalized treatment strategies. Though some of the novel aspects identified are incremental, they contribute to the growing body of evidence in this domain and underscore the need for continued biomarker exploration in OAB research.

## Figures and Tables

**Table 1:** Demographic and clinical baseline parameters compared in the female control and OAB groups.

	CTR Group (*n* = 23)	OAB Group (*n* = 33)	*p* Value
Demographic and Serum Analysis			
Age (years), mean ± SD (range)	56.62 ± 5.46 (50.0–72.0)	69.49 ± 10.14 (50.0–86.0)	<0.0001 *
BMI (kg/m^2^)	27.05 ± 5.30	27.36 ± 4.80	0.8247
Systolic BP (mmHg)	121.17 ± 12.80	127.76 ± 15.05	0.0930
eGFR (mL/min/1.73 m^2^)	96.39 ± 16.45	75.33 ± 18.08	<0.0001 *
HbA1c (%)	5.50 ± 0.44	5.70 ± 0.70	0.1581
Fasting glucose (mmol/L)	5.34 ± 0.74	5.97 ± 1.62	0.0885
HOMA-IR	2.03 ± 0.97	4.01 ± 3.71	0.0288 *
Total Chol/HDL	3.39 ± 1.15	3.09 ± 0.72	0.2400
Questionnaires’ scores			
OABS (0–28)	13.04 ± 4.89	21.03 ± 6.58	<0.0001 *
ICIQ (0–22)	4.13 ± 3.69	9.36 ± 4.49	<0.0001 *
IIQ7 (0–100)	2.08 ± 4.92	31.93 ± 24.04	<0.0001 *
Voiding diary parameters			
24-h frequency	8.74 ± 2.43	10.75 ± 3.07	0.0112 *
Daytime frequency	8.13 ± 2.16	8.97 ± 2.34	0.1786
Night frequency	0.61 ± 0.78	1.78 ± 1.43	0.0007 *
24-h voided volume (mL)	2526.8 ± 2230.7	1779.2 ± 809.5	0.0821
Nocturnal urine volume (mL)	437.1 ± 291.02	429.7 ± 283.7	0.9229
Mean voided volume (mL)	310.2 ± 291.02	174.4 ± 78.24	0.0132 *
Maximum voided volume (mL)	473.5 ± 190.13	329.6 ± 134.6	0.0016 *

Data are presented as mean ± SD for variables compared with a parametric *t*-test; statistically significant differences are reported (* *p* < 0.05). All numbers in the table are rounded to two decimals.

**Table 2:** Urinary biomarker levels of control and OAB groups and in pre- vs. post-treatment in the OAB group.

	CTR Group (*n* = 23)	*p* Value ^a^	OAB Baseline (*n* = 10)	OAB 3-Months (*n* = 10)	*p* Value ^b^
proNGF/Cr (ng/mg)	0.5261 (0.3519, 0.7497)	0.5536	0.5838 (0.2724, 0.7499)	0.6786 (0.4465, 1.774)	0.0488 *
NGF/Cr (pg/mg)	1.668 (1.173, 3.22)	0.0034 *	0.5671 (0.3697, 1.002)	0.778 (0.595, 2.43)	0.0273 *
proNGF/NGF ratio mol/mol	268.9 (164.3, 370.6)	0.0002 *	712.1 (658.0, 809.2)	645.1 (560.6, 735.6)	0.1309
NGF/proNGF ratio mol/mol	0.003 (0.002, 0.006)	0.0002 *	0.0015 (0.0012, 0.0017)	0.0015 (0.0013, 0.0017)	0.1055
proBDNF/Cr (pg/mg)	640.8 (364.5, 2028)	0.3454	502.4 (220.8, 1433)	662.9 (360.3, 1256)	0.6953
BDNF/Cr (pg/mg)	10.32 (6.71, 28.54)	0.3045	11.49 (4.869, 44.37)	16.82 (7.384, 44.62)	0.4922
proBDNF/BDNF ratio mol/mol	27,972 (15,710, 44,408)	0.0095 *	18,301 (14,718, 24,649)	18,089 (14,402, 25,753)	0.3223
BDNF/proBDNF ratio mol/mol	0.00004 (0.00002, 0.00006)	0.0076 *	0.00005 (0.00004, 0.00007)	0.00005 (0.00003, 0.00007)	0.2891
p75^ECD^/Cr (ng/mg)	2.614 (2.155, 3.012)	0.1843	2.804 (2.005, 6.029)	4.329 (3.167, 7.714)	0.2324

Data are presented as median (interquartile range, Q1, Q3). Statistically significant differences are reported (* *p* < 0.05). a Compared with the non-parametric Mann–Whitney U test. b Compared with the Wilcoxon matched pairs signed rank test.

## Data Availability

The data that support the findings of this study are available on request from the corresponding author. The data are not publicly available due to privacy or ethical restrictions.

## Abbreviations

The following abbreviations are used in this manuscript:

BDNF	Brain-Derived Neurotrophic Factor
eGFR	Estimated Glomerular Filtration Rate
HOMA-IR	Homeostatic Model Assessment of Insulin Resistance
ICIQ-SF	International Consultation on Incontinence Questionnaire-Short Form
IIQ	Incontinence Impact Questionnaire
NGF	Nerve Growth Factor
OAB	Overactive Bladder Syndrome
OABSS	Overactive Bladder Symptom Score
p75^ECD^	p75 Extracellular Domain
proBDNF	Proform of Brain-Derived Neurotrophic Factor
proNGF	Proform of the Nerve Growth Factor

## Appendix A

Results adjusted to covariates maintained similar trends, emphasizing a relationship between aging, renal function, metabolic disturbances, and neurotrophin imbalance in the context of OAB.

**Table A1:** This is a table caption. Urinary NGF and proteolytic enzymes were compared in the female control and OAB groups, considering confounders.

	Confounders	CTR Group (*n* = 23)	OAB Group (*n* = 33)	*p* Value
proNGF/Cr (ng/mg)	Age	0.562 (0.372–0.752)	0.689 (0.540–0.837)	0.341
	HOMA-IR	0.533 (0.339–0.728)	0.675 (0.518–0.832)	0.270
	eGFR	0.553 (0.373–0.733)	0.695 (0.552–0.838)	0.252
NGF/Cr (pg/mg)	Age	2.201 (1.688–2.716)	1.107 (0.703–1.510)	0.003 *
	HOMA-IR	1.989 (1.455–2.523)	1.043 (0.612–1.474)	0.010 *
	eGFR	1.882 (1.383–2.380)	1.319 (0.924–1.715)	0.102
proNGF/NGF ratio (mol/mol)	Age	252.69 (19.346–486.03)	820.18 (637.57–1002.79)	<0.001 *
	HOMA-IR	332.67 (85.548–579.80)	846.93 (647.31–1046.55)	0.003 *
	eGFR	382.60 (152.59–612.61)	733.57 (551.022–916.12)	0.029 *
NGF/proNGF ratio (mol/mol)	Age	0.005 (0.004–0.006)	0.002 (0.001–0.003)	0.006 *
	HOMA-IR	0.005 (0.004–0.006)	0.002 (0.001–0.003)	<0.001 *
	eGFR	0.004 (0.003–0.005)	0.003 (0.002–0.004)	0.060
proBDNF/Cr (ng/mg)	Age	1339.54 (776.55–1902.54)	795.70 (343.89–1247.51)	0.176
	HOMA-IR	1602.51 (1014.97–2190.05)	719.46 (231.27–1207.65)	0.028 *
	eGFR	1311.42 (775.95–1846.89)	815.30 (380.44–1250.16)	0.184
BDNF/Cr (pg/mg)	Age	25.17 (12.856–37.492)	26.48 (16.595–36.365)	0.881
	HOMA-IR	21.03 (9.421–32.646)	25.46 (15.815–35.112)	0.567
	eGFR	21.40 (9.520–33.281)	29.11 (19.463–38.759)	0.350
proBDNF/BDNF ratio (mol/mol)	Age	25.55 (19.232–31.870)	19.68 (14.613–24.755)	0.193
	HOMA-IR	31.47 (25.309–37.632)	17.79 (12.674–22.913)	0.002 *
	eGFR	28.02 (21.733–34.306)	17.96 (12.858–23.068)	0.024 *
BDNF/proBDNF ratio (mol/mol)	Age	0.070 (0.035–0.105)	0.090 (0.062–0.118)	0.422
	HOMA-IR	0.038 (0.006–0.071)	0.093 (0.066–0.120)	0.014 *
	eGFR	0.052 -0.017–0.087)	0.103 (0.074–0.131)	0.041 *
p75^ECD^/Cr (ng/mg)	Age	3.168 (2.133–4.202)	3.552 (2.722–4.383)	0.600
	HOMA-IR	2.747 (1.721–3.773)	3.575 (2.723–4.428)	0.229
	eGFR	2.896 (1.886–3.906)	3.741 (2.921–4.562)	0.229

Data are presented as estimated marginal means (95% CI) for variables compared with ANCOVA. Statistical significance was further adjusted for multiple comparisons using Bonferroni correction. Statistical significance reported at *p* < 0.05 *. Abbreviations: CTR: control; NGF: nerve growth factor; BDNF: brain-derived neurotrophic factor; Cr: creatinine; HOMA-IR: Homeostatic Model Assessment for Insulin Resistance; eGFR: estimated glomerular filtration rate; p75^ECD^: neurotrophin receptor p75 extracellular domain.
